# The RNA helicase DHX35 functions as a co-sensor for RIG-I-mediated innate immunity

**DOI:** 10.1371/journal.ppat.1012379

**Published:** 2024-07-22

**Authors:** Yuan Qiao, Shan Zhu, Ning Yang, Shan-Shan Zou, Bao Gao, Jing Wu, Chunyan Liu, Xiaoping Li, Yong-Jun Liu, Jingtao Chen

**Affiliations:** 1 Cancer Center, The First Hospital of Jilin University, Changchun, China; 2 Laboratory for Tumor Immunology, The First Hospital of Jilin University, Changchun, China; The Ohio State University, UNITED STATES OF AMERICA

## Abstract

RNA helicases are involved in the innate immune response against pathogens, including bacteria and viruses; however, their mechanism in the human airway epithelial cells is still not fully understood. Here, we demonstrated that DEAH (Asp-Glu-Ala-His) box polypeptide 35 (DHX35), a member of the DExD/H (Asp-Glu-x-Asp/His)-box helicase family, boosts antiviral innate immunity in human airway epithelial cells. DHX35 knockdown attenuated the production of interferon-β (IFN-β), IL6, and CXCL10, whereas DHX35 overexpression increased their production. Upon stimulation, DHX35 was constitutively expressed, but it translocated from the nucleus into the cytosol, where it recognized cytosolic poly(I:C) and poly(dA:dT) via its HELICc domain. Mitochondrial antiviral signaling protein (MAVS) acted as an adaptor for DHX35 and interacted with the HELICc domain of DHX35 using amino acids 360–510. Interestingly, DHX35 interacted with retinoic acid-inducible gene 1 (RIG-I), enhanced the binding affinity of RIG-I with poly(I:C) and poly(dA:dT), and formed a signalsome with MAVS to activate interferon regulatory factor 3 (IRF3), NF-κB-p65, and MAPK signaling pathways. These results indicate that DHX35 not only acted as a cytosolic nucleic acid sensor but also synergized with RIG-I to enhance antiviral immunity in human airway epithelial cells. Our results demonstrate a novel molecular mechanism for DHX35 in RIG-I-mediated innate immunity and provide a novel candidate for drug and vaccine design to control viral infections in the human airway.

## Author summary

RIG-I plays an important role in defending against RNA viral infections, such as SARS-CoV-2, influenza virus, and vesicular stomatitis virus (VSV). DHX35 is a member of the Asp-Glu-x-Asp/His (DExD/H)-box helicase family. This study revealed a novel function of DHX35 in regulating activation of the RIG-I signaling pathway. DHX35 translocates from the nucleus into cytosol where it recognizes cytosolic nucleic acids upon stimulation, and interacts with MAVS to activate downstream signaling pathways. More importantly, DHX35 interacts with RIG-I to enhance its binding affinity to nucleic acids. Taken together, our findings demonstrate a novel molecular mechanism of RIG-I-mediated innate immunity in human airway epithelial cells.

## Introduction

The lungs are the site of air exchange with the outside environment, where many viruses, bacteria, and other airborne microorganisms exist. Infection with these pathogens can cause an array of human diseases, such as asthma, pulmonary abscess, chronic obstructive pneumonia disease, and pulmonary tuberculosis [1]. The respiratory epithelium not only provides a physical barrier but also recognizes the nucleic acids of these pathogens to help prevent infection [2–7] and is thus critical to activate antiviral immunity.

Innate immunity is the first line of defense against pathogens. A variety of germline-encoded pattern recognition receptors (PRRs), such as Toll-like receptors (TLRs) and retinoic acid-inducible gene 1 (RIG-I)-like receptors, can sense and recognize pathogen-associated molecular patterns to trigger the production of interferons (IFNs), inflammatory cytokines, and chemokines which eliminate viruses [8,9]. TLRs are the earliest PRRs discovered in innate immunity [10]. There are 13 TLRs, of which TLR3, TLR7/8, and TLR9 recognize dsRNA, ssRNA, and unmethylated CpG DNA in the endosomal compartment, respectively. They then recruit adaptor proteins—TRIF for TLR3 and MyD88 for TLR7/8/9—to phosphorylate interferon regulatory factor (IRF)3/7, leading to the production of IFNs, inflammatory cytokines, and chemokines [11]. For nucleic acids in the cytosol, the most important cytosolic RNA sensors are RIG-I and melanoma differentiation-associated protein 5 (MDA5). RIG-I recognizes 5´-triphosphate (5´-ppp) and short-chain dsRNA with high affinity, while MDA5 recognizes long-chain dsRNA with weaker affinity via the C-terminal domain [12–15]. After binding with dsRNA, RIG-I and MDA5 undergo a conformational change and recruit mitochondrial antiviral signal protein (MAVS) to activate TBK1 and IRF3, leading to the production of IFNs, inflammatory cytokines, and chemokines [16,17]. cGAS is a universal DNA sensor that catalyzes the synthesis of cGAMP after binding with cytosolic DNA [18], and it is subsequently sensed by stimulator of interferon genes (STING), also known as ERIS, MITA, or MYPS. STING is phosphorylated and interacts with TBK1 to activate IRF3, leading to the production of IFNs, inflammatory cytokines, and chemokines [19–22].

In addition to the PRRs mentioned above, DEAD/DEAH (DEAD/H)-box helicases, which are involved in RNA metabolism, gene expression, and programmed cell death, also play important roles in antiviral immunity [23–25]. For example, DEAD-box helicase 1 (DDX1) binds with dsRNA and forms a complex with DDX21 and DDX36 to promote type I interferon via TRIF in myeloid dendritic cells (mDCs) [26]. DHX9, DHX15, and DHX33 sense cytosolic dsRNA and produce type I interferon through MAVS in mDCs [27–29]. DHX9 also activates NF-κB via CpG-B DNA-recognition and DHX36 selectively binds to CpG-A DNA to promote IFN-α production in human plasmacytoid dendritic cells (pDCs) [30]. DDX41 interacts with intracellular DNA and the secondary messenger cyclic di-GMP (c-di-GMP) or cyclic di-AMP (c-diAMP) to activate the interferon response through STING in mDCs [31]. DDX46 recruits the m^6^A demethylase, ALKBH5, to erase the m^6^A modification of MAVS, TRAF3, and TRAF6 transcripts, leading to their retention in the nucleus, which promotes RNA and DNA replication in macrophages [32]. In human airway epithelial cells, only DHX29 was identified as a co-sensor for RIG-I to promote the activation of antiviral immunity [3], but the relationship of RIG-I and DHX29 in antiviral immunity remains unclear. Thus, the roles and mechanisms of DEAD/H-box helicases in human airway epithelial cell antiviral immunity require further elucidation.

DHX35 belongs to the family of DEAD/H-box helicases. It has been reported that high DHX35 expression predicts poor prognosis in patients with hepatocellular carcinoma [33]. DHX35 is epigenetically upregulated by treatment with selenium, which antagonizes cadmium-induced breast carcinogenesis [34]. Furthermore, DHX35 knockdown suppresses the replication of myxoma virus, a double-stranded DNA (dsDNA) virus, in human cancer cells [35,36]. However, the mechanisms underlying DHX35’s involvement in antiviral immunity in human airway epithelial cells remain unclear.

In this study, we investigated the role of DHX35 in the RIG-I/MAVS signaling pathway in human airway epithelial cells. Our mechanistic studies suggest that DHX35 acts as a co-sensor of RIG-I for the recognition of RIG-I agonist poly(I:C) and poly(dA:dT), playing an essential role in the innate immune response to cytosolic poly(I:C) and poly(dA:dT) in human airway epithelial cells.

## Results

### DHX35 positively regulates the production of IFN-β, IL6, and CXCL10 in human airway epithelial cells

**Fig 1:**
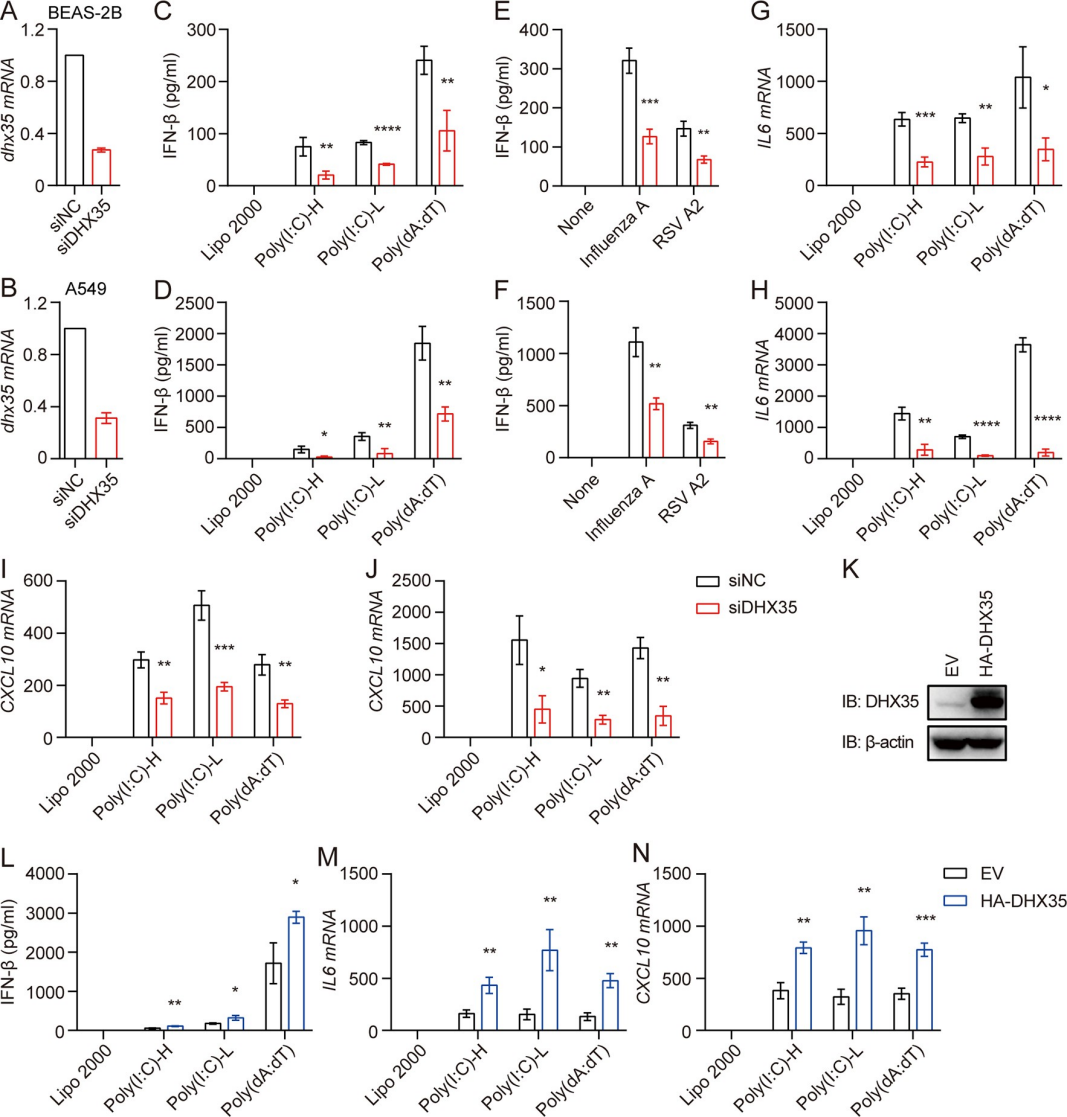
DHX35 promotes antiviral innate immunity in human airway epithelial cells. (A and B) BEAS-2B (A) and A549 (B) cells were transfected with siRNAs for DHX35. After 48 h, RNA was extracted and qPCR was performed to detect the knockdown efficiency of DHX35. (C and D) DHX35 knockdown BEAS-2B (C) and A549 (D) cells were transfected with poly(I:C)-H, poly(I:C)-L, and poly(dA:dT). Supernatants were harvested 18 h later and secretion of IFN-β was measured by ELISA. (E and F) DHX35 knockdown BEAS-2B (E) and A549 (F) cells were infected with influenza A and RSV A2 virus. Supernatants were harvested 18 h later and secretion of IFN-β was measured by ELISA. (G–J) DHX35 knockdown BEAS-2B (G and I) and A549 (H and J) cells were transfected with poly(I:C)-H, poly(I:C)-L, and poly(dA:dT). RNA was extracted 18 h later and qPCR was performed to detect the expression of IL6 (H and H) and CXCL10 (I and J). (K) A549 cells were transfected with HA-DHX35. After 48 h, cells were lysed and subjected to SDS-PAGE to detect DHX35 expression; β-actin was used as a loading control. (L–N) DHX35-overexpressed A549 cells were transfected with poly(I:C)-H, poly(I:C)-L, and poly(dA:dT). Supernatants were harvested 18 h later and secretion of IFN-β was measured by ELISA (L). RNA was extracted and qPCR was performed to detect IL6 (M) and CXCL10 expression (N). All data are plotted as the mean ± SD. Results are representative of three independent experiments. *P < 0.05, **P < 0.01, ***P < 0.001, ****P < 0.0001 vs. the corresponding control. HA-DHX35: HA-tagged DHX35; NC: negative control; EV: empty vector.

To investigate the role of DHX35 in antiviral immunity, we knocked down the expression of DHX35 using small interfering RNA (siRNA) in human airway epithelial cell lines BEAS-2B and A549. The results showed that the expression of DHX35 was significantly decreased at the mRNA level (Fig 1A and 1B).

We and others have reported that human airway epithelial cells can sense cytoplasmic synthetic nucleic acid analogs, such as 5′-ppp-dsRNA, long poly(I:C) (poly(I:C)-H), short poly(I:C) (poly(I:C)-L), poly(dA:dT), or RNA viruses via the RIG-I/MAVS signaling pathway [3,8]. Thus, we stimulated cells with poly(I:C)-H, poly(I:C)-L, poly(dA:dT), influenza A, and RSV A2 virus and detected the production of IFN-β. We found that IFN-β production was significantly reduced (Fig 1C–1F) and the expression of IL6 and CXCL10 was dramatically decreased (Fig 1G–1J).

To confirm the role of DHX35 in human airway epithelial cell antiviral immunity, we overexpressed DHX35 in A549 cells (Fig 1K). IFN-β production was significantly increased by poly(I:C)-H, poly(I:C)-L, and poly(dA:dT) (Fig 1L), and the expression levels of IL6 and CXCL10 were significantly enhanced (Fig 1M and 1N). Taken together, these results indicate that DHX35 boosts innate immunity in the human airway epithelial system.

### DHX35 bound cytosolic nucleic acids via the HELICc domain

**Fig 2:**
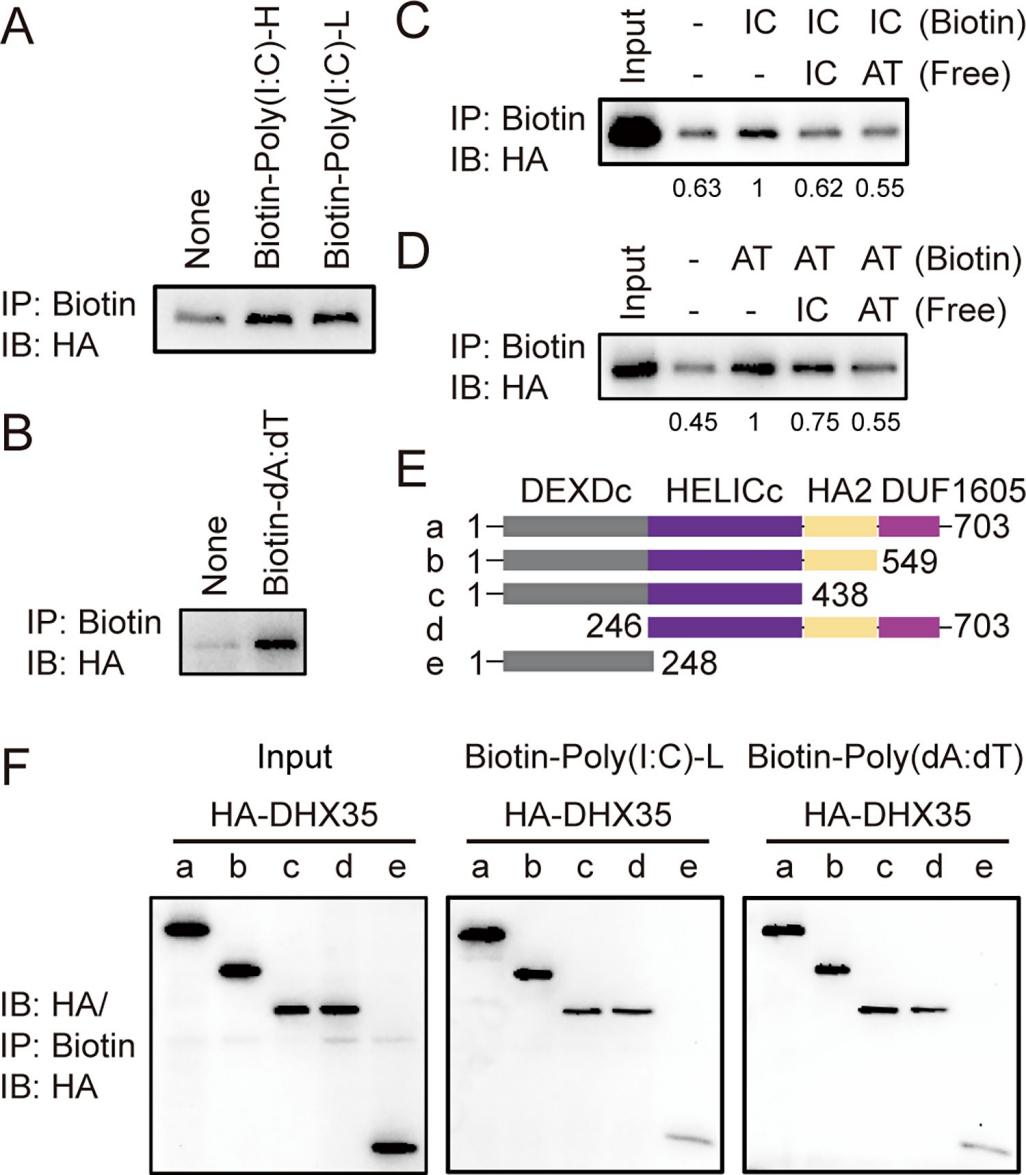
DHX35 directly binds cytosolic nucleic acids via the HELICc domain. (A and B) HEK293T cells were transfected with HA-DHX35. Twenty-four hours later, cells were lysed and cell lysates were mixed with biotinylated poly(I:C)-H, poly(I:C)-L, and poly(dA:dT) for 1 h. Then, NeutrAvidin beads were added and incubated. After 4 h, the beads were washed with lysis buffer and the protein was eluted and subjected to western blotting with HA antibody. (C and D) HA-DHX35-overexpressed HEK293T cell lysates were mixed with biotinylated poly(I:C) and poly(dA:dT) with or without nonbiotinylated free poly(I:C) and poly(dA:dT) and were subjected to the same procedure as described in A. (E) Schema of DHX35 domain. (F) HEK293T cells were transfected with full-length and truncated HA-DHX35 plasmids and were subjected to the same procedure as described in A. Results are representative of at least three independent experiments. IC: poly(I:C); AT: poly(dA:dT). HA-DHX35: HA-tagged DHX35.

Numerous studies have reported that RNA helicases sense cytosolic nucleic acids [3,26–31,37–39]. To investigate whether DHX35 is a cytosolic nucleic acid sensor, an in vitro pull-down assay was performed to detect the interaction of DHX35 with poly(I:C) and poly(dA:dT) in HA-tagged DHX35-overexpressed cell lysates. The results showed that DHX35 bound to biotinylated poly(I:C)-HMW, poly(I:C)-LMW, and poly(dA:dT) (Fig 2A and 2B). To clarify the specificity of this binding, competition assays were performed using non-labeled free poly(I:C) and poly(dA:dT). The results showed that non-labeled poly(I:C) and poly(dA:dT) could compete with both nucleic acids (Fig 2C and 2D). Thus, DHX35 can bind to poly(I:C) and poly(dA:dT).

To determine which DHX35 domain was involved in binding to poly(I:C) and poly(dA:dT), a schematic diagram of the DHX35 domain was plotted according to data from the UCSC website (http://genome.ucsc.edu/) (Fig 2E). Then, each HA-tagged truncated mutation construct was overexpressed in 293T cells, and an in vitro pull-down assay was performed. The results showed that removal of the DHX35 HELICc domain abolished binding of DHX35 with poly(I:C) and poly(dA:dT) (Fig 2F), indicating that the HELICc domain of DHX35 was indispensable for binding between DHX35 and cytosolic nucleic acids.

### DHX35 translocated from the nucleus into the cytoplasm after stimulation

**Fig 3:**
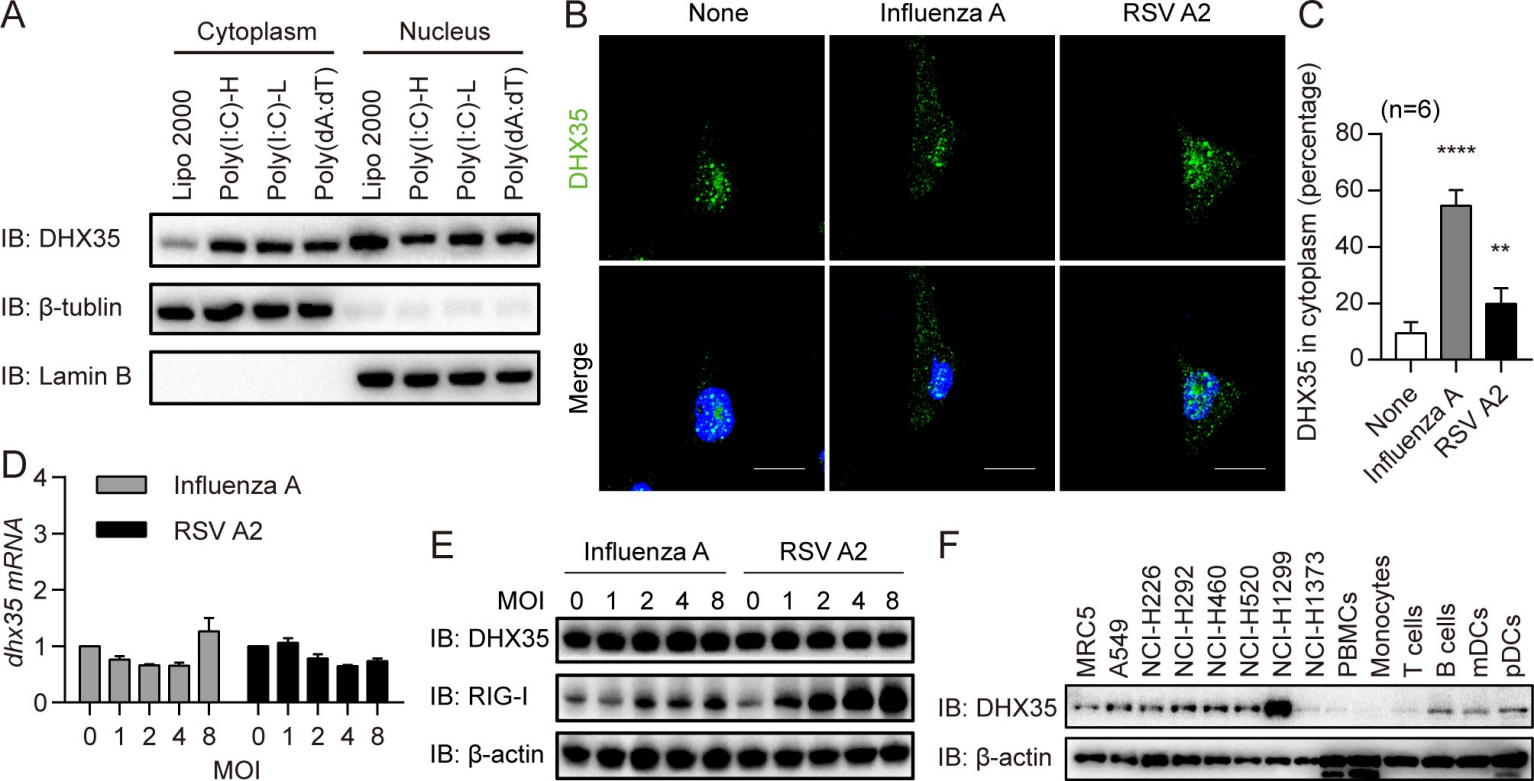
DHX35 translocates from the nucleus into the cytosol after stimulation. (A) BEAS-2B cells were transfected with poly(I:C)-H, poly(I:C)-L, and poly(dA:dT). Protein in the cytosol and nucleus was extracted 6 h later and subjected to SDS-PAGE to detect the expression of DHX35, β-tubulin, and Lamin B. Data are representative of three independent experiments. (B) BEAS-2B cells were infected with influenza A and RSV A2 virus for 6 h and then DHX35 expression was detected by immunofluorescence. (C) The location of DHX35 was analyzed using Image J software and differences were calculated. (D) BEAS-2B cells were infected with influenza A and RSV A2 virus for 6 h at MOI of 1, 2, 4, and 8. Then, RNA was extracted and the expression of DHX35 was detected by qPCR. (E) BEAS-2B cells were infected with influenza A and RSV A2 virus for 6 h at MOI of 1, 2, 4, and 8. Then, cells were lysed and cell lysates were subjected to SDS-PAGE to detect the expression of DHX35. Results are representative of three independent experiments. (F) Cells from MRC5, A549, NCI-H226, NCI-H460, NCI-H520, NCI-H1299, NCI-H1373, PBMCs, monocytes, T cells, B cells, mDCs, and pDCs were lysed, and cell lysates were subjected to SDS-PAGE to detect DHX35 expression.

Viral RNA/DNA is injected into the host cell cytoplasm after infection, whereas DHX35 is primarily located in the nucleus at a steady state, according to the database (https://www.genecards.org/cgi-bin/carddisp.pl?gene=DHX35#localization). Therefore, determining the location and expression of DHX35 before and after stimulation is critical. Cells were transfected with poly(I:C)-H, poly(I:C)-L, and poly(dA:dT), and the expression of DHX35 in the cytoplasm and nucleus was detected. The results showed that DHX35 expression increased in the cytoplasm and decreased in the nucleus (Figs 3A and S1A). To further confirm the location of DHX35, cells were infected with RNA virus. The results showed that DHX35 was more located in the cytoplasm than in the nucleus (Figs 3B, 3C, S1B and S1C). However, there was no difference in the total expression of DHX35 after stimulation (Figs 3D, 3E and S1D–S1F).

Moreover, DHX35 expression in several human airway epithelial cells, peripheral blood-derived mononuclear cells (PBMCs), PBMC derived monocytes, T cells, B cells, mDCs, and pDCs were detected. The results showed that DHX35 was expressed in most cell types (Fig 3F). Taken together, these results indicate that DHX35 is constitutively expressed and translocated into the cytosol, where it interacts with cytosolic nucleic acids upon infection in human airway epithelial cells.

### MAVS acted as an adaptor protein for DHX35

**Fig 4:**
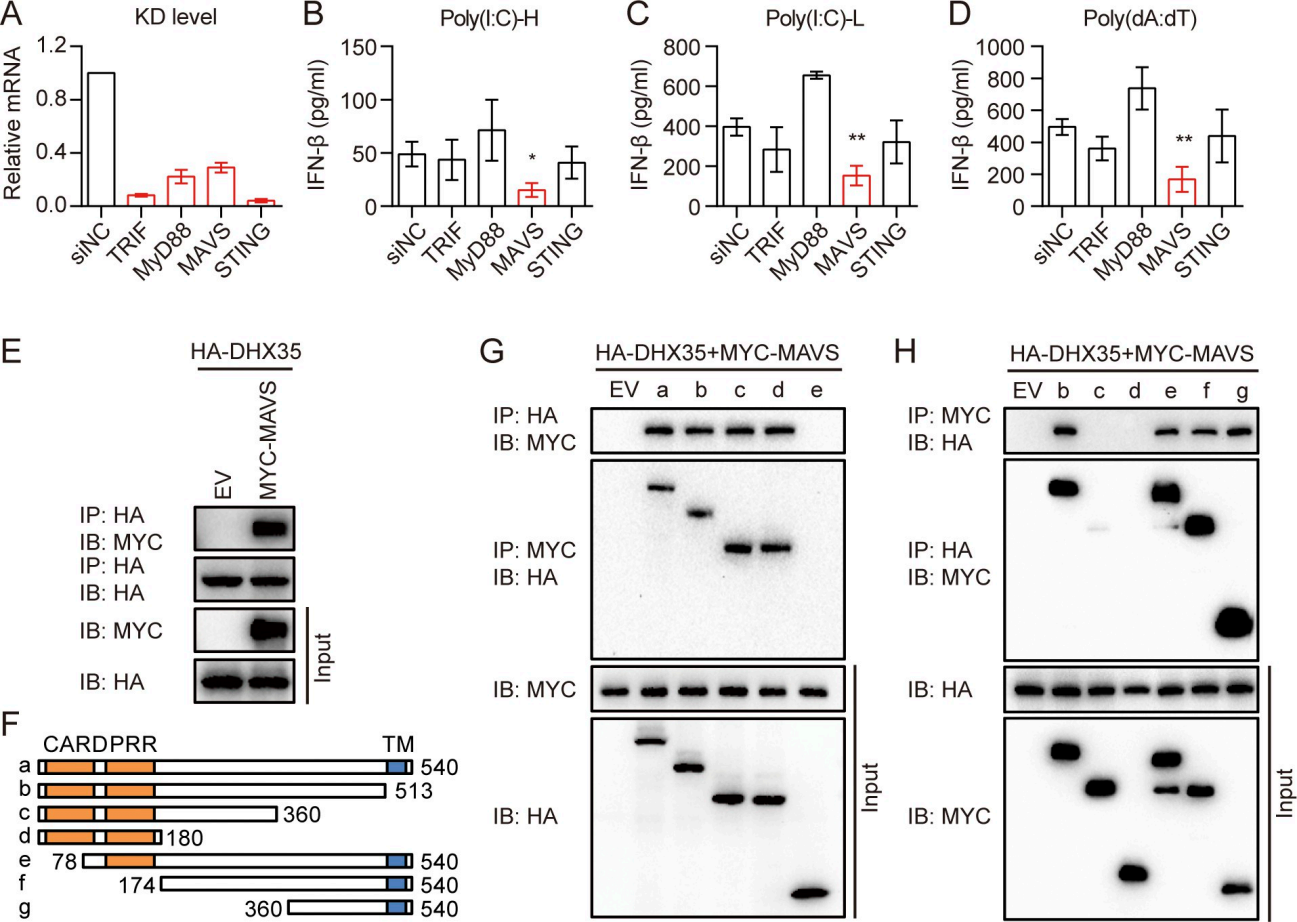
MAVS acts as the adaptor protein for DHX35. (A) A549 cells were transfected with TRIF, MyD88, MAVS, and STING siRNAs. After 48 h, RNA was extracted and qPCR was performed to detect the knockdown efficiency. (B–D) TRIF, MyD88, MAVS, and STING knockdown A549 cells were transfected with poly(I:C)-H, poly(I:C)-L, and poly(dA:dT). Supernatants were harvested 18 h later and IFN-β secretion was measured by ELISA. (E) HEK293T cells were transfected with HA-DHX35 and empty vector (EV) or MYC-MAVS. Cells were lysed and total protein was used for CO-IP experiments 24 h later. (F) HEK293T cells were transfected with full-length or truncated HA-DHX35 and MYC-MAVS. Cells were lysed and total protein was used to perform CO-IP experiments 24 h later. (G) Schema of MAVS domain. (H) HEK293T cells were transfected with full-length or truncated MYC-MAVS and HA-DHX35. Cells were lysed and total protein was used to perform CO-IP experiments 24 h later. All data are plotted as the mean ± SD. Results are representative of three independent experiments. *P < 0.05, **P < 0.01 vs. the corresponding control. HA-DHX35: HA-tagged DHX35; MYC-MAVS: MYC-tagged MAVS; NC: negative control; EV: empty vector.

To determine the adaptor protein of DHX35, we knocked down the expression of TRIF, MyD88, MAVS, and STING, which have been reported as adaptors in innate immunity, using siRNA in A549 cells. We found that these molecules were significantly inhibited at the mRNA level (Fig 4A). IFN-β production was significantly reduced by poly(I:C)-H, poly(I:C)-L, and poly(dA:dT) when MAVS was silenced (Fig 4B–4D). To further determine whether MAVS plays a role in DHX35-mediated innate immunity, we performed a CO-IP experiment and found that DHX35 interacted with MAVS (Fig 4E). These results suggest that MAVS acted as an adaptor protein for DHX35.

To identify which DHX35 domain interacts with MAVS, we transfected HEK293T cells with full-length and truncated HA-DHX35 and MYC-tagged MAVS (MYC-MAVS), then performed CO-IP. The results showed that DHX35 interacted with MAVS via its HELICc domain (Fig 4F and 4G). Similar experiments were performed using full-length and truncated MYC-MAVS and HA-DHX35 to determine which MAVS domain interacts with DHX35; our results revealed that MAVS interacted with DHX35 via its 360–510 amino acids (Fig 4H).

### DHX35 synergized with RIG-I to sense cytosolic nucleic acids

**Fig 5:**
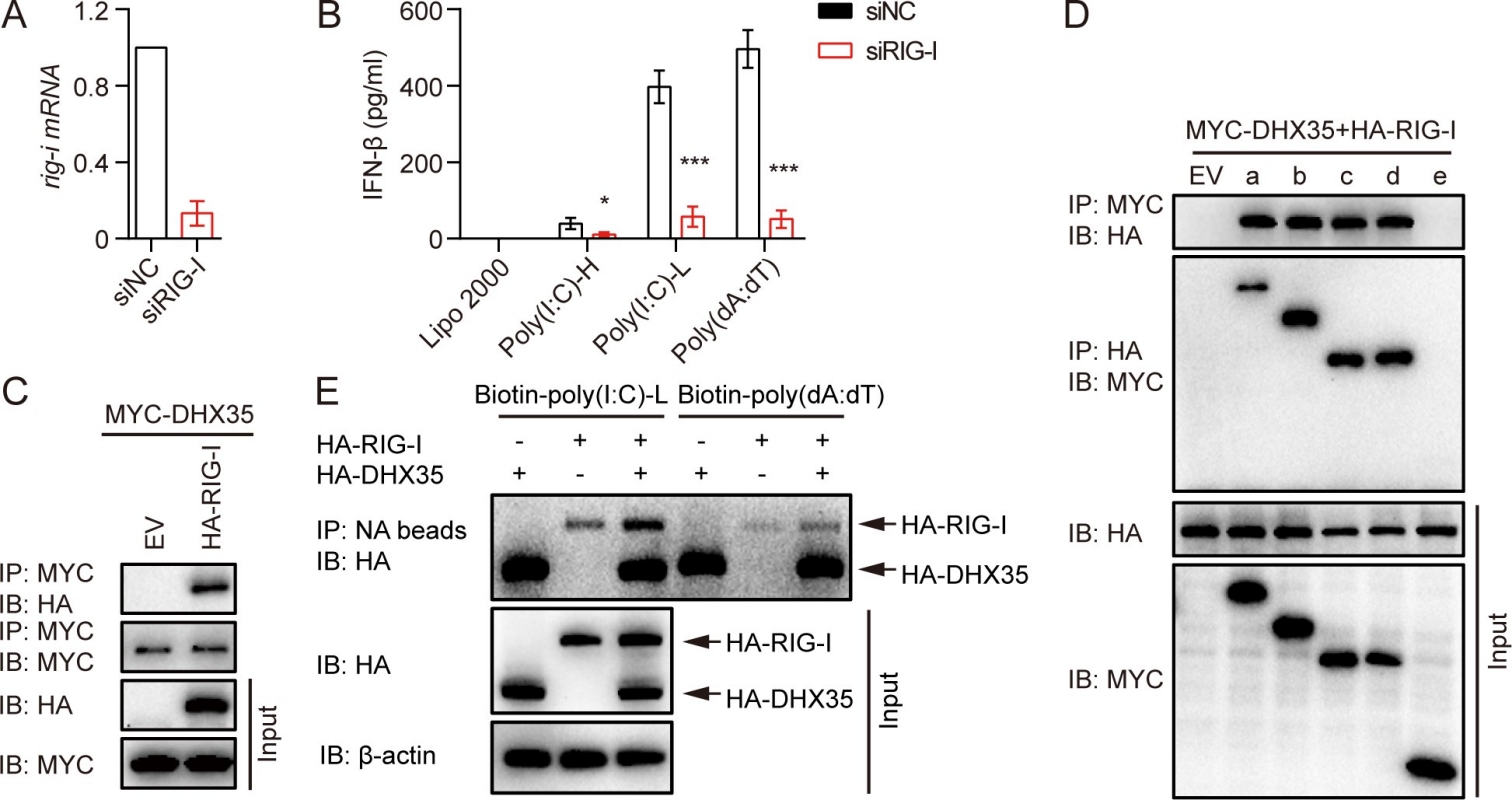
DHX35 interacts with RIG-I to promote the production of IFN-β. (A) A549 cells were transfected with siRNA for RIG-I. The expression of RIG-I was examined by qPCR 48 h later. (B) RIG-I knockdown A549 cells were transfected with poly(I:C)-H, poly(I:C)-L, and poly(dA:dT) for 18 h, and the production of IFN-β in the supernatants was measured by ELISA. (C) HEK293T cells were transfected with EV or HA-RIG-I and MYC-DHX35 for 24 h. Then, cells were lysed and total protein was used for CO-IP experiments. (D) HEK293T cells were transfected with full-length or truncated MYC-DHX35 and HA-DHX35. Cells were lysed and total protein was used to perform CO-IP experiments 24 h later. (E) HEK293T cells were transfected with HA-RIG-I, HA-DHX35, or HA-RIG-I, and HA-DHX35 for 24 h. Cells were lysed and total protein was coprecipitated with biotin-poly(I:C) or biotin-poly(dA:dT) for in vitro pull-down analysis. All data are plotted as the mean ± SD. Results are representative of three independent experiments. *P < 0.05, ***P < 0.001 vs. the corresponding control. HA-DHX35: HA-tagged DHX35; MYC-DHX35: MYC-tagged DHX35; HA-RIG-I, HA-tagged RIG-I; NC: negative control; EV: empty vector.

A previous study reported that RIG-I is crucial in sensing cytosolic poly(I:C) and poly(dA:dT) in human epithelial cells [3]. Thus, we knocked down the expression of RIG-I in A549 cells, which resulted in significantly reduced IFN-β secretion induced by poly(I:C)-H, poly(I:C)-L, and poly(dA:dT) (Fig 5A and 5B). To elucidate the relationship between DHX35 and RIG-I, we performed CO-IP experiments and found that DHX35 interacted with RIG-I (Fig 5C). Further analysis revealed that DHX35 interacted with RIG-I via its HELICc domain (Fig 5D).

It has been previously reported that DHX29 enhances the RNA recognition of MDA5 [40]; therefore, we considered whether DHX35 enhanced the binding affinity of RIG-I with poly(I:C) and poly(dA:dT). 293T cells were co-transfected with HA-DHX35 and HA-tagged RIG-I (HA-RIG-I), and an in vitro pull-down assay was performed. The results showed that DHX35 not only sensed poly(I:C) and poly(dA:dT) but also enhanced the binding affinity of RIG-I with poly(I:C) and poly(dA:dT) (Fig 5E), indicating that DHX35 acted as a co-sensor of RIG-I to enhance cytosolic poly(I:C)-and poly(dA:dT)-induced innate immunity in human airway epithelial cells.

### DHX35, RIG-I, and MAVS formed a signalsome that activated downstream signaling pathways

**Fig 6:**
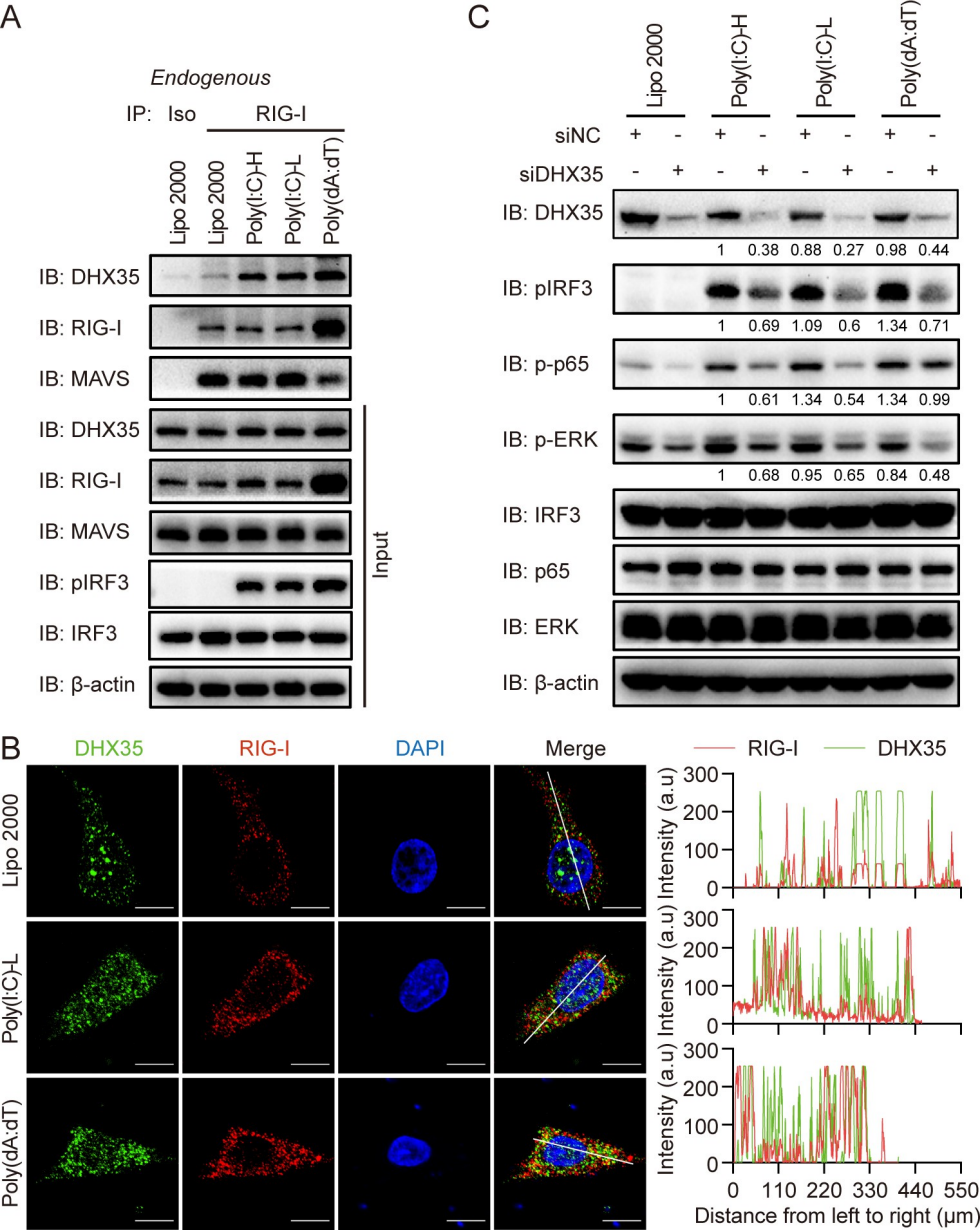
DHX35, RIG-I, and MAVS form a signalosome to activate downstream signaling pathways. (A) A549 cells were transfected with poly(I:C)-H, poly(I:C)-L, and poly(dA:dT). Cells were lysed 6 h later and RIG-I antibody was added to the total protein for endogenous CO-IP experiments. (B) A549 cells were transfected with poly(I:C) and poly(dA:dT) for 6 h and then subjected to immunofluorescence staining (left). The co-localization of DHX35 and RIG-I was analyzed using Image J software (right). Results are representative of at least five independent fields. (C) A549 cells were transfected with DHX35 siRNA for 48 h, then cells were transfected with poly(I:C)-H, poly(I:C)-L, and poly(dA:dT). Cells were lysed 6 h later and subjected to SDS-PAGE to detect the expression of DHX35, pIRF3, p-p65, pERK, IRF3, p65, and ERK. β-actin was used as a loading control. Gray value was analyzed using Image J software. NC: negative control.

Our results suggested that DHX35, RIG-I, and MAVS were involved in poly(I:C)- and poly(dA:dT)-induced innate immunity in human airway epithelial cells. Next, we investigated whether DHX35, RIG-I, and MAVS formed a signalsome after stimulation. The endogenous CO-IP results showed that RIG-I interacted with DHX35 and MAVS after transfection with poly(I:C)-H, poly(I:C)-L, and poly(dA:dT) (Fig 6A). In addition, DHX35 translocated into the cytosol from the nucleus and colocalized with RIG-I after stimulation with poly(I:C) and poly(dA:dT) (Fig 6B), further indicating that DHX35, RIG-I, and MAVS form a signalsome after stimulation with poly(I:C) and poly(dA:dT) in human airway epithelial cells.

Finally, we confirmed the role of DHX35 in poly(I:C)- and poly(dA:dT)-induced downstream signaling pathways in human airway epithelial cells. Our results showed that knockdown of DHX35 impaired poly(I:C)-H-, poly(I:C)-L-, and poly(dA:dT)-induced phosphorylation of NF-κB-p65, IRF3, and ERK in human airway epithelial cells (Fig 6C). Taken together, our results suggest that DHX35 acted as a co-sensor and synergized with RIG-I to boost the innate immunity in human airway epithelial cells.

## Discussion

Recent studies have reported that RNA helicases are implicated in antiviral immunity [23]. They are not only involved in sensing cytosolic nucleic acids directly [26–31], but also act as coreceptors for RLR-MAVS and cGAS-STING signaling pathways to defend against various viruses, such as hepatitis B virus, hepatitis C virus, herpes simplex virus, vesicular stomatitis virus (VSV), encephalomyocarditis virus (EMCV), and human immunodeficiency virus [3,40–43]. In this study, we provide compelling evidence that the DEAD/H-box helicase DHX35 functions as a RIG-I co-sensor and plays a critical role in RIG-I-mediated antiviral innate immunity. Our results showed that DHX35 not only recognized cytosolic poly(I:C)-H, poly(I:C)-L, and poly(dA:dT) via its HELICc domain, but also enhanced the binding affinity of RIG-I with poly(I:C) and poly(dA:dT) to promote activation of the RIG-I/MAVS signaling pathway. Our results extend the number of identified RNA helicase family members that are involved in antiviral immunity; moreover, this study is the first to report that DHX35 is a molecule that promotes RIG-I mediated antiviral immunity in human airway epithelial cells.

DHX35 is highly expressed in human hepatocellular carcinoma and breast cancer cells and promotes carcinogenesis [33,34]. A study reported that DHX35 knockdown suppressed myxoma virus (a dsDNA virus) replication in human cancer cells [35]; however, its expression and location are unknown in human airway epithelial cells. In this study, we found that DHX35 is expressed in all human airway epithelial cell lines, including MRC5, A549, NCI-H226, NCI-H292, NCI-H460, NCI-H520, NCI-H1299, and NCI-H1373, as well as in human PBMCs, monocytes, T cells, B cells, cDCs, and pDCs. Although there was no difference in the expression of DHX35 after infection with RNA virus and transfection with the synthetic nucleic acid analogs poly(I:C) and poly(dA:dT), DHX35 translocated into the cytosol from the nucleus where it recognized cytosolic poly(I:C) and poly(dA:dT) and interacted with RIG-I and MAVS to enhance the production of IFN-β, IL6, and CXCL10 in human airway epithelial cells, albeit the mechanism of DHX35 translocation is unknown.

RNA helicases regulate antiviral immunity via various mechanisms. One study reported that DHX29 co-senses cytosolic poly(I:C) and poly(dA:dT) in human airway epithelial cells and fibroblasts [3]. Another study found that DHX29 sensed poly(I:C)-HMW, but not poly(I:C)-HMW or poly(dA:dT), and enhanced MDA5-dsRNA binding affinity to promote EMCV-specific antiviral immunity [40]. DHX15 selectively binds viral RNA to promote RIG-I ATP hydrolysis and the activation of downstream signaling pathways [42]. DDX6 binds viral RNA and interacts with RIG-I to stimulate RIG-I-mediated induction of IFN-β in response to influenza B virus infection [43]. In our study, we report a novel mechanism whereby DHX35 not only recognized cytosolic poly(I:C) and poly(dA:dT) directly, but also enhanced the binding affinity of RIG-I with poly(I:C) and poly(dA:dT) to promote the activation of downstream signaling pathways in human airway epithelial cells.

A previous study reported that DHX35 knockdown suppressed replication of myxoma virus, a member of the dsDNA poxvirus family, in human cancer cells, including A549 cells [35,44]. However, in contrast to these findings, our results show that knockdown of DHX35 significantly reduced the production of IFN-β, IL6 and CXCL10, and inhibited the activation of downstream signaling pathways after infection with RNA virus and transfection with cytosolic poly(I:C) and poly(dA:dT), whereas overexpression of DHX35 increased the production of IFN-β, IL6 and CXCL10 in human airway epithelial cells. These results suggest that DHX35 may play opposing roles in viral RNA and DNA infection. Thus, it is necessary to distinguish between DNA or RNA virus infections during drug and vaccine design for viral infection.

In conclusion, we demonstrate the mechanisms underlying the role of DHX35 as a co-sensor in the RIG-I-mediated innate immune response to cytosolic poly(I:C)-H, poly(I:C)-L, poly(dA:dT), and RNA viruses in human airway epithelial cells. Our results enhance our understanding of various infections and immune pathologies, providing a novel candidate for drug and vaccine design for the control of RNA viruses and AT-rich DNA virus infections in human airways.

## Materials and methods

### Cell culture

All cell lines were obtained from the American Type Culture Collection (ATCC). BEAS-2B cells were maintained in Bronchial Epithelial Cell Basal Medium (Lonza, CC-3171). HEK293T, MRC5, and NCI-H1373 cells were maintained in Dulbecco’s modified Eagle’s medium, while A549, NCI-H226, NCI-H292, NCI-H460, NCI-H520, and NCI-H1299 cells were maintained in RPMI-1640. All media contained 10% heat-inactivated fetal bovine serum (TransGen, FS401-02) and 1% penicillin–streptomycin (TransGen, FG101-01). All cell lines were mycoplasma-free and authenticated using short tandem repeat identification by the Microread Company (Beijing, China). Human PBMCs were purified with Ficoll-Paque PLUS (GE Healthcare, 17144003). Then, monocytes, T cells, B cells, mDCs, and pDCs were labeled and sorted with BD Influx (BD Biosciences) using antibodies against CD3, CD4, CD8, CD14, CD16, CD19, CD56, and CD11c (BD Biosciences).

### Plasmid constructs

To construct overexpression plasmids, the cDNA of DHX35 and MAVS were amplified by reverse transcriptase polymerase chain reaction (RT-PCR) using total RNA extracted from A549 cells as templates and subcloned into pCMV-HA (Clontech, 635690) or pCMV-MYC (Clontech, 635689) using the In-Fusion PCR cloning kit (TransGen, CU101-01) with Kpn I (New England Biolabs, R3142). Truncated forms were generated based on pCMV-HA-DHX35 and pCMV-MYC-MAVS. The sequence fidelity of the full-length and truncated cDNA clones was verified by DNA sequencing. The primers used were as follows:

DHX35-a (amino acids 1–2109 bp) sense: 5´-CCGAGATCTCTCGAGGTACCGCTGCGCCCGTGGGACCGGTGAAGTT-3´, antisense: 5´-ATCCCCGCGGCCGCGGTACCTCACGGGTCCTGGACCTTGGCCCTTTT-3´; DHX35-b (amino acids 1–1647 bp) sense: 5´-CCGAGATCTCTCGAGGTACCGCTGCGCCCGTGGGACCGGTGAAGTT-3´, antisense: 5´-ATCCCCGCGGCCGCGGTACCTCAATTGAGCATAGTGAGGTGGTCGCC-3´; DHX35-c (amino acids 1–1344 bp) sense: 5´-CCGAGATCTCTCGAGGTACCGCTGCGCCCGTGGGACCGGTGAAGTT-3´, antisense: 5´-ATCCCCGCGGCCGCGGTACCTCAAATTCCTAGTGCTTTCAGCTGCAGG-3´; DHX35-d (amino acids 738–2109 bp) sense: 5´-CCGAGATCTCTCGAGGTACCTTTTATCTACAAAGTCCTGTTCCAG-3´, antisense: 5´-ATCCCCGCGGCCGCGGTACCTCACGGGTCCTGGACCTTGGCCCTTTT-3´; DHX35-e (amino acids 1–744 bp) sense: 5´-CCGAGATCTCTCGAGGTACCGCTGCGCCCGTGGGACCGGTGAAGTT-3´, antisense: 5´-ATCCCCGCGGCCGCGGTACCTCAATAAAAGATATCCACCGG-3´; MAVS-a (amino acids 1–1620 bp) sense: 5´-CCGAGATCTCTCGAGGTACCCCGTTTGCTGAAGACAAGACC-3´, antisense: 5´-ATCCCCGCGGCCGCGGTACCCTAGTGCAGACGCCGCCGGTACA-3´; MAVS-b (amino acids 1–1542 bp) sense: 5´-CCGAGATCTCTCGAGGTACCCCGTTTGCTGAAGACAAGACC-3´, antisense: 5´-ATCCCCGCGGCCGCGGTACCCTACCCAGGTGAGGGCCTGT-3´; MAVS-c (amino acids 1–1080 bp) sense: 5´-CCGAGATCTCTCGAGGTACCCCGTTTGCTGAAGACAAGACC-3´, antisense: 5´-ATCCCCGCGGCCGCGGTACCCTAGGATGGCACCATGCCAGCAC-3´; MAVS-d (amino acids 1–540 bp) sense: 5´-CCGAGATCTCTCGAGGTACCCCGTTTGCTGAAGACAAGACC-3´, antisense: 5´-ATCCCCGCGGCCGCGGTACCCTAGGAGGACTCCAGGGGGCCA-3´; MAVS-e (amino acids 235–1620 bp) sense: 5´-CCGAGATCTCTCGAGGTACCGAGCTAGTTGATCTCGCGGACGAAG-3´, antisense: 5´-ATCCCCGCGGCCGCGGTACCCTAGTGCAGACGCCGCCGGTACA-3´; MAVS-f (amino acids 523–1620 bp) sense: 5´-CCGAGATCTCTCGAGGTACCGGCCCCCTGGAGTCCTCCTCTGA-3´, antisense: 5´-ATCCCCGCGGCCGCGGTACCCTAGTGCAGACGCCGCCGGTACA-3´; MAVS-g (amino acids 1081–1620 bp) sense: 5´-CCGAGATCTCTCGAGGTACCAAAGTGCCTACTAGCATGGTGCTCA-3´, antisense: 5´-ATCCCCGCGGCCGCGGTACCCTAGTGCAGACGCCGCCGGTACA-3´.

#### *Virus*, *infection*, *and* nucleic acid *transfection*

Influenza A and RSV A2 strains were kindly provided by Professor Chunlai Jiang from the College of Life Science at Jilin University. For virus infection, cells were incubated with influenza A and RSV A2 virus. For nucleic acids transfection, cells were transfected with 5 mg/mL poly(I:C)-HMW (InvivoGen, tlrl-pic), poly(I:C)-LMW (InvivoGen, tlrl-picw), and poly(dA:dT) (InvivoGen, tlrl-patn) using 5 μL/mL of Lipofectamine 2,000 (Invitrogen, 11668019) according to manufacturer’s instructions.

#### RNA interference

Transfection of small interfering RNA (siRNA) specific for RIG-I, TRIF, MAVS, and STING was performed as previously described [8]. Human DHX35 siRNA was purchased from Invitrogen (1299001). Human MyD88 siRNA was purchased from Sigma-Aldrich (EHU029771). All siRNAs were transfected using Lipofectamine RNAiMAX (Invitrogen, 13778150), according to the manufacturer’s instructions. Forty-eight hours after transfection, the knockdown level was assessed by real-time quantitative PCR (qPCR), and the cells were used for subsequent experiments.

#### RNA extraction and qPCR

Total RNA was isolated using the EasyPure RNA kit (TransGen, ER101-01), according to the manufacturer’s instructions. Reverse transcription was performed using the EasyScript FirstStrand cDNA Synthesis SuperMix Kit (TransGen, AE301-03). RT-PCR was performed using SYBR Green Supermix (Roche, 4913914001). All primers were synthesized by Jilin Comate Co., Ltd. (Changchun, China). The sequences of the primers used were as follows:

DHX35 sense: 5´-GCTGCTGTTACAGTTGCAGG-3´, antisense: 5´-CTGGTCGGTGCAGTCATCAAA-3´; IL6 sense: 5´-ACTCACCTCTTCAGAACGAATTG-3´, antisense: 5´-CCATCTTTGGAAGGTTCAGGTTG-3´; CXCL10 sense: 5´-GTGGCATTCAAGGAGTACCTC-3´, antisense: 5´-TGATGGCCTTCGATTCTGGATT-3´; TRIF sense: 5´-GCCAGCAACTTGGAAATCAGC-3´, antisense: 5´-GGGGTCGTCACAGAGCTTG-3´; MyD88 sense: 5´-GGCTGCTCTCAACATGCGA-3´, antisense: 5´-CTGTGTCCGCACGTTCAAGA-3´; MAVS sense: 5´-CAGGCCGAGCCTATCATCTG-3´, antisense: 5´-GGGCTTTGAGCTAGTTGGCA-3´; STING sense: 5´-CCAGAGCACACTCTCCGGTA-3´, antisense: 5´-CGCATTTGGGAGGGAGTAGTA-3´; RIG-I sense: 5´-CTGGACCCTACCTACATCCTG-3´, antisense: 5´-GGCATCCAAAAAGCCACGG-3´; GAPDH sense: 5´-GGAGCGAGATCCCTCCAAAAT-3´, antisense: 5´-GGCTGTTGTCATACTTCTCATGG-3´. GAPDH was used as the housekeeping gene to normalize the amount of cDNA. The relative expression was calculated using the 2^(-ΔΔCt)^ method.

### ELISA

Cells were stimulated for 18 h, then, culture supernatants were harvested, and human IFN-β secretion was assessed using an ELISA kit (PBL Interferon Source, 41410–1), according to the manufacturer’s instructions. The absorbance was measured at 450 nm using a microplate reader (BioTek). The concentrations of the samples were determined according to the standard curve.

### Western blotting

Cells were lysed on ice for 30 min with cell lysis buffer (Cell Signaling Technology, 9803) supplemented with a Protease Inhibitor Cocktail (MCE, HY-K0010). Lysates were centrifuged at 13,000 g for 15 min, and the supernatants were collected. The protein concentration was measured using a Bradford assay kit (Thermo Fisher Scientific, 23225). Forty micrograms of total protein were subjected to sodium dodecyl sulfate-polyacrylamide gel electrophoresis (SDS-PAGE) and transferred to polyvinylidene difluoride (PVDF) membranes. The membranes were blocked with 5% non-fat milk in PBS containing 0.1% Tween 20 for 1 h. The membranes were incubated with the following primary antibodies: DHX35 (Abcam; ab235366; 1:1,000), β-actin (TransGen; HC201-01; 1:1,000), HA (Cell Signaling Technology; 3724; 1:1,000), β-tubulin (Cell Signaling Technology; 2146; 1:1,000), Histone H3 (Cell Signaling Technology; 4499; 1:1,000), pIRF3 (Immunoway; 1:1,000), IRF3 (Cell Signaling Technology; 11904; 1:1,000), MYC (Cell Signaling Technology; 2276; 1:1,000), RIG-I (Cell Signaling Technology; 3743; 1:1,000), MAVS (Santa Cruz Biotechnology; sc-166583; 1:1,000), p-p65 (Cell Signaling Technology; 3033, 1:1,000), p65 (Cell Signaling Technology; 8242, 1:1,000), pERK (Cell Signaling Technology; 4370, 1:1,000), and ERK (Cell Signaling Technology; 4696, 1:1,000) at 4°C overnight, followed by 1:5,000 secondary antibodies (PerkinElmer, 130549 and 10148784) for 1 h at room temperature. The bands were visualized using a chemiluminescence system (ECL Advance; Amersham Biosciences). Gray value was analyzed using Image J software (NIH).

### In vitro RNA/DNA pull-down assay

The poly(I:C) and poly(dA:dT) were labeled with biotin using the RNA 3´ End Biotinylation Kit (Thermo Fisher Scientific, 20160) and Biotin 3’ End DNA Labeling Kit (Thermo Fisher Scientific, 89818), respectively, according to the manufacturer’s instructions. HEK293T cells transfected with the indicated plasmids were lysed with RIPA buffer (Cell Signaling Technology) supplemented with a Protease Inhibitor Cocktail (MCE). Lysates were incubated with biotinylated poly(I:C)/poly(dA:dT) for 1 h at 4°C and then incubated with High Capacity NeutrAvidin Agarose (Thermo Fisher Scientific, 29201) for another 4 h at 4°C. The bound complexes were washed three times with lysis buffer and analyzed by immunoblotting with HA antibody.

### Nuclear and cytoplasmic extraction

Nuclear and cytoplasmic extractions were performed using Nuclear and Cytoplasmic Extraction Reagents (Thermo Scientific, 78833) according to the manufacturer’s instructions. Briefly, the cells were transfected with poly(I:C)-HMW, poly(I:C)-LMW and poly(dA:dT) for 6 h. The cells were then washed with PBS and harvested using trypsin-EDTA. Cell pellets were sequentially lysed with ice-cold CER I and CER II reagents and centrifuged at maximum speed for 5 min at 4°C. The supernatants (cytoplasmic extract) were collected, and the insoluble (pellet) fractions were lysed with ice-cold NER reagent for 40 min. Lysis was centrifuged at maximum speed for 10 min at 4°C, and the supernatants (nuclear extract) were collected for SDS-PAGE.

### Confocal microscopy

Cells were cultured overnight on glass coverslips and stimulated for 6 h. The cells were then washed with PBS and fixed with 4% paraformaldehyde for 10 min. After washing three times with PBS, the cells were permeabilized with 0.1% Triton X-100 and blocked with 5% BSA in PBS for 1 h at room temperature. Then, they were incubated with DHX35 (Invitrogen, PA5-66518, 2 μg/mL) and RIG-I (Invitrogen, 700366, 1:100) overnight at 4°C. Cells were washed three times with PBS, labeled with DAPI for 5 min, and visualized using an Olympus confocal microscope (Olympus; FV3000) at the Institute of Immunology, the First Hospital of Jilin University. All pictures were analyzed using Image J software (NIH).

To calculate the percentage of DHX35 in cytosol, the total fluorescence intensity of the cells and the fluorescence intensity of nuclei were analyzed using ImageJ software. Cytoplasmic fluorescence intensity was obtained by subtracting nuclear fluorescence intensity from total fluorescence intensity. The percentage of cytoplasmic fluorescence was calculated by dividing the cytoplasmic fluorescence intensity by the total fluorescence intensity. More than five random fields of cells were analyzed and used for statistical analysis.

The quantification of co-localization by immunofluorescence was determined using ImageJ/Fiji software (N.I.H., Bethesda, MD) [45].

### Co-immunoprecipitation (CO-IP) assays using transfected HEK293T cells

HEK293T cells were transfected with the indicated plasmids using Lipofectamine 2000 (Invitrogen) according to the manufacturer’s instructions for 24 h and lysed in cell lysis buffer (Cell Signaling Technology) supplemented with Protease Inhibitor Cocktail (MCE). Cell lysates were immunoprecipitated overnight at 4°C using anti-HA magnetic beads (Thermo Fisher Scientific, 88836) or anti-MYC magnetic beads (Thermo Fisher Scientific, 88843). The magnetic beads were washed three times with TENT buffer. The precipitates were immunoblotted with HA and MYC antibodies.

### Co-immunoprecipitation (CO-IP) assays using poly(I:C)/poly(dA:dT)-transfected cells

The cells were seeded in a 6-well plate. The next day, these cells were transfected with poly(I:C) and poly(dA:dT) for 4 h and then lysed in cell lysis buffer supplemented with Protease Inhibitor Cocktail. Lysates were immunoprecipitated with RIG-I antibody or isotype control (Cell Signaling Technology, 3900S) overnight at 4°C. Protein A Magnetic Beads (Cell Signaling Technology, 73778S) was added and incubated for 4 h. Sepharose beads were washed three times with TENT buffer, and the precipitates were analyzed using standard immunoblotting procedures.

### Statistical analysis

Statistical differences between experimental and control groups were determined by the Student’s t-test using GraphPad Prism 7 software (GraphPad Software, San Diego, CA, USA). Statistical significance was set at P < 0.05. All data are represented as the mean ± standard deviation (SD) from three independent experiments.

The numerical data used in all figures are included in S1 Data.

## Supporting information

S1 Data Excel spreadsheet containing, in separate sheets, the underlying numerical data for Figs 1A, 1B, 1C, 1D, 1E, 1F, 1G, 1H, 1I, 1J, 1M, 1N, 3C, 3D, 4A, 4B, 4C, 4D, 5A, 5B, 6B, S1C and S1D. (XLSX)

S1 Fig DHX35 translocates from the nucleus into the cytosol after stimulation. (A) A549 cells were transfected with poly(I:C)-H, poly(I:C)-L, and poly(dA:dT). Six hours later, protein in the cytosol and nucleus was extracted and subjected to SDS-PAGE to detect the expression of DHX35, β-tubulin, and Lamin B. Data are representative of three independent experiments. (B) A549 cells were infected with influenza A and RSV A2 virus for 6 h and then DHX35 expression was detected by immunofluorescence. (C) the location of DHX35 was analyzed using Image J software. (D) A549 cells were infected with influenza A and RSV A2 virusfor 18 h. Then, RNA was extracted and qPCR were performed to detect the expression of DHX35. (E) A549 cells were infected with influenza A and RSV A2 virus for 6 h, Then, cells were lysed and subjected to SDS-PAGE to detect the expression of DHX35. Data are representative of three independent experiments. (F) A549 cells were transfected with poly(I:C)-H, poly(I:C)-L, and poly(dA:dT) for 6 h, Then, cells were lysed and subjected to SDS-PAGE to detect the expression of DHX35. Data are representative of three independent experiments.
(TIF)
