# Supplementary figures and images for: The RNA helicase DHX35 functions as a co-sensor for RIG-I-mediated innate immunity
Source: PLoS Pathog. 2024 Jul 22;20(7):e1012379. doi: 10.1371/journal.ppat.1012379 (PMC11262647; doi:10.1371/journal.ppat.1012379)

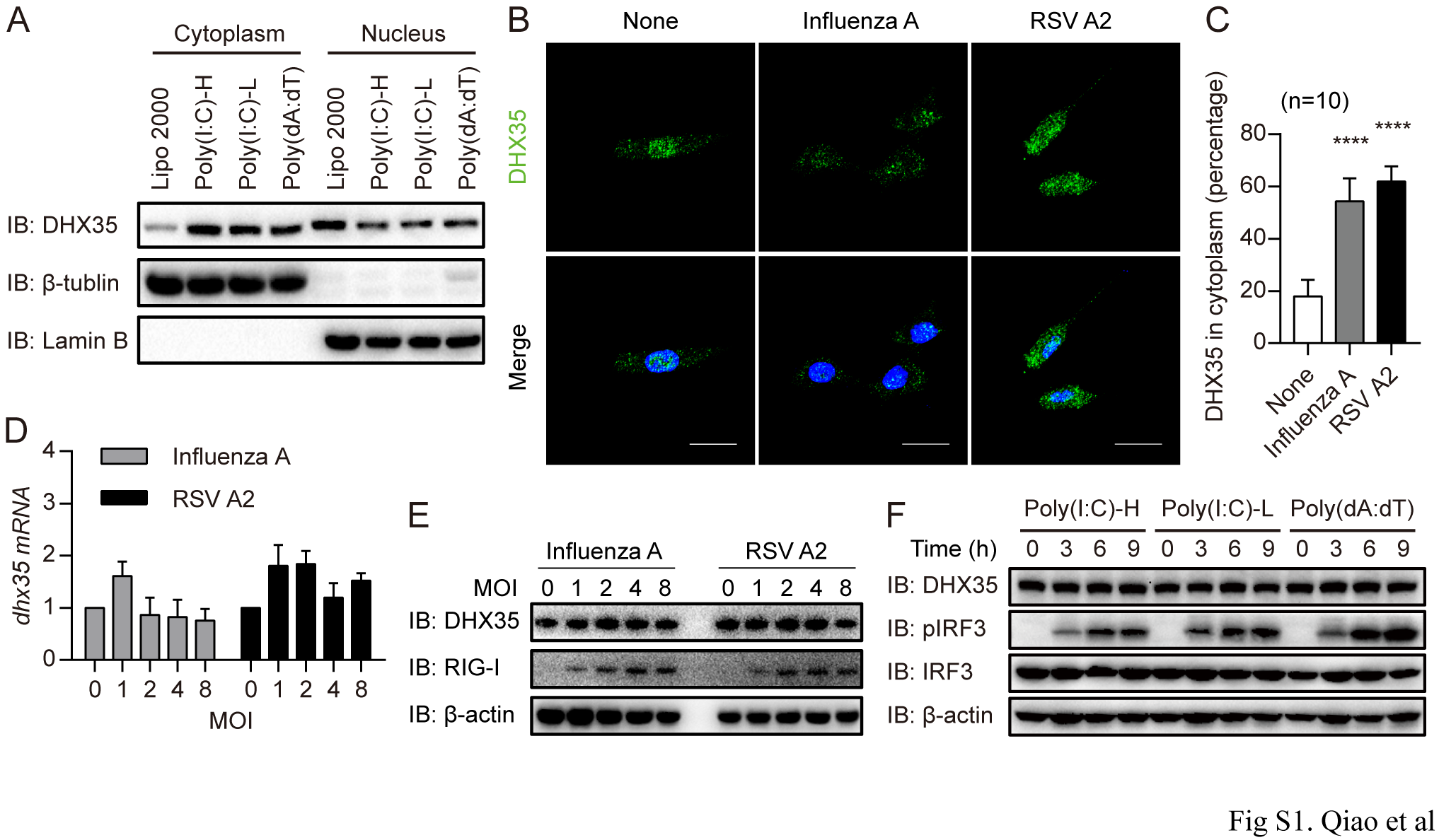

Supplement: S1 Fig — (A) A549 cells were transfected with poly(I:C)-H, poly(I:C)-L, and poly(dA:dT). Six hours later, protein in the cytosol and nucleus was extracted and subjected to SDS-PAGE to detect the expression of DHX35, β-tubulin, and Lamin B. Data are representative of three independent experiments. (B) A549 cells were infected with influenza A and RSV A2 virus for 6 h and then DHX35 expression was detected by immunofluorescence. (C) the location of DHX35 was analyzed using Image J software. (D) A549 cells were infected with influenza A and RSV A2 virusfor 18 h. Then, RNA was extracted and qPCR were performed to detect the expression of DHX35. (E) A549 cells were infected with influenza A and RSV A2 virus for 6 h, Then, cells were lysed and subjected to SDS-PAGE to detect the expression of DHX35. Data are representative of three independent experiments. (F) A549 cells were transfected with poly(I:C)-H, poly(I:C)-L, and poly(dA:dT) for 6 h, Then, cells were lysed and subjected to SDS-PAGE to detect the expression of DHX35. Data are representative of three independent experiments. (TIF) [file ppat.1012379.s002.tif]
